# Supplementary material for: Long-Term Incidence of Total Knee Arthroplasty after Open Reduction and Internal Fixation of Proximal Tibial and Distal Femoral Fractures: A Nationwide Cohort Study
Source: J Clin Med. 2021 Dec 2;10(23):5685. doi: 10.3390/jcm10235685 (PMC8658582; doi:10.3390/jcm10235685)
Supplement: Supplementary file 1 [file jcm-10-05685-s001.zip › jcm-1476878-supplementary.pdf]

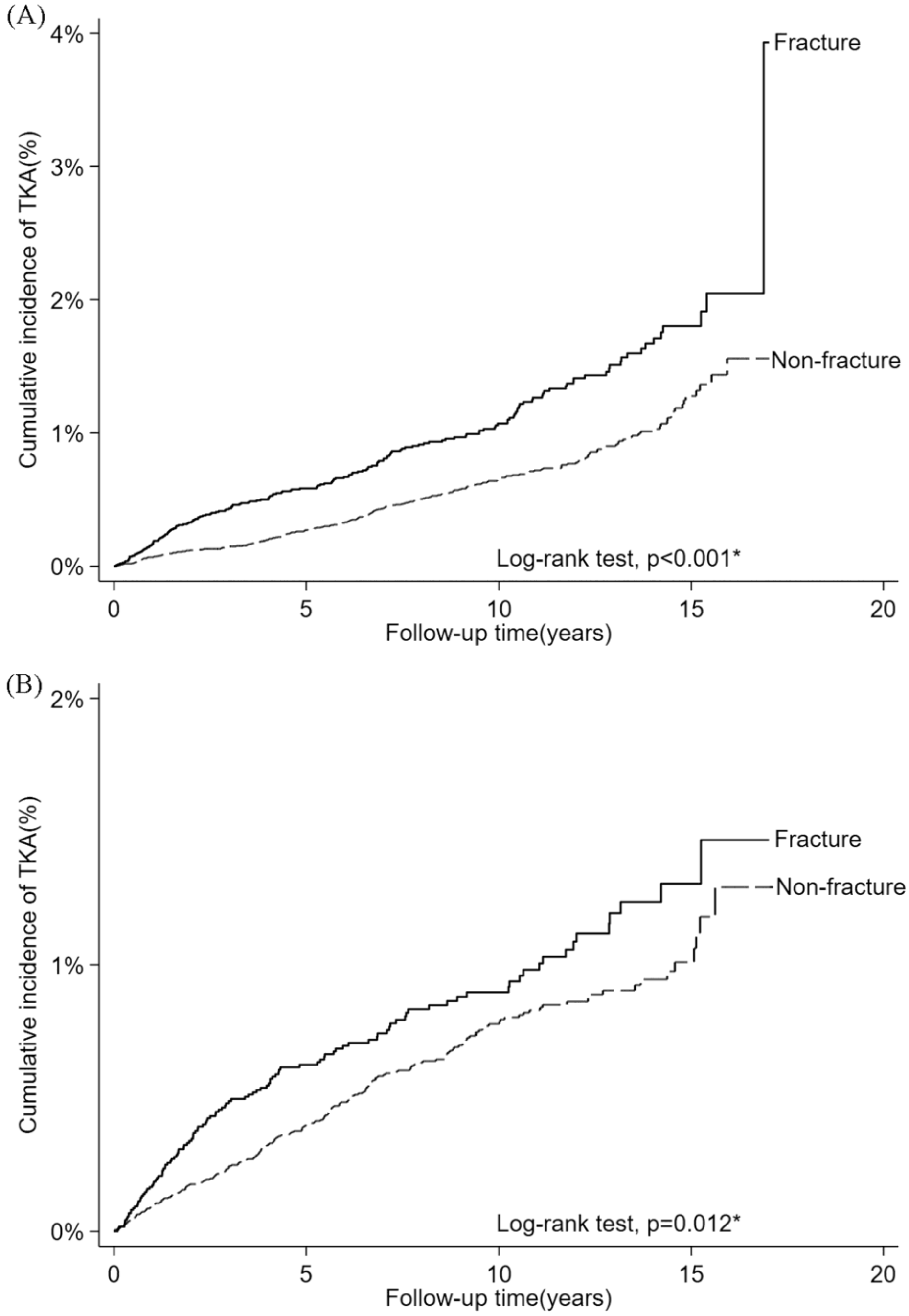

Figure S1. The proportion of subjects receiving TKA of the fracture group and the non-fracture group according to age, sex, and index date at a 1:2 ratio. (**A**) The 20–65-year subgroups of the ORIF and non-fracture groups. (**B**) The male subgroups of the ORIF and non-fracture groups.

Table S1. ICD9 and ICD10 diagnosis codes.

Diagnosis	ICD-9-CM	ICD-10-CM
Hypertension	401–405	I10,I11,I12,I13,I15
Diabetes Mellitus	250	E10,E11,E13
Hyperlipidemia	272.0–272.9	E78
Chronic renal failure	585.0–585.9	N184,N185,N186,N189
Coronary artery disease	410–414	I20,I21,I22,I24,I25
Cerebrovascular disease	430–438	I60,I61,I62,I63,I64,I65,I66,I67,I68,I69,G45,G46
Alcohol-induced mental disorders	291.0–291.9	F1014,F1019,F1024,F1026,F1029,F1027,F1094,F1096,F1097,F1099,F10121,F10150,F10151,F10159,F10180,F10181,F10182,F10188,F10221,F10230,F10231,F10232,F10239,F10250,F10251,F10259,F10280,F10281,F10282,F10288,F10920,F10921,F10929,F10950,F10951,F10959,F10980,F10981,F10982,F10988
Alcohol dependence syndrome	303.0–303.9	F1020,F1021,F10120,F10129,F10220,F10229
Drug dependence	304.0–304.9	F11,F12,F13,F14,F15,F16,F18,F19
Chronic liver disease	571.0–571.9	K70,K73,K74,K754,K760,K769,K7581,K7689
Iron deficiency anemia	280.0–280.9	D50
Depression	296.2, 296.3, 296.82, 300.4, 309.0, 309.1, 311.0–311.9	F30,F32,F40,F41,F43,F44,F68
Dementia	290, 294.1, 294.2, 331.0–331.9	F01,F02,F03,G30,G31
Peripheral vascular disease	440,443.90	I70,I739

Table S2. Baseline characteristics and comorbidity according to age, sex, and index year at a 1:2 ratio.

	Age/sex/index year matching (1:2)		
	**Control (*n* = 65,254)**	**Case (*n* = 32,627)**	***p*-value**
Age (y/o)	58.44±19.92	58.44±19.92	1.000
Age Group2			1.000
<65 y/o	36,492(55.9%)	18,246(55.9%)	
≧65 y/o	28,762(44.1%)	14,381(44.1%)	
Male(%)	33,808(51.8%)	16,904(51.8%)	1.000
Comorbidity			
HTN	18,304(28.1%)	10,037(30.8%)	<0.001*
DM	7801(12.0%)	5756(17.6%)	<0.001*
Hyperlipidemia	7110(10.9%)	3441(10.6%)	0.097
Chronic renal failure	1125(1.7%)	1017(3.12%)	<0.001*
CAD	5628(8.6%)	2880(8.8%)	0.290
CVA	4266(6.5%)	3134(9.6%)	<0.001*
Alcohol-induced mental disorders	35(0.1%)	84(0.3%)	<0.001*
Alcohol dependence syndrome	41(0.1%)	131(0.4%)	<0.001*
Drug dependence	16(<0.1%)	61(0.2%)	<0.001*
Chronic liver disease	2763(4.2%)	1709(5.2%)	<0.001*
Iron deficiency anemia	460(0.7%)	385(1.2%)	<0.001*
Depression	1743(2.7%)	1426(4.4%)	<0.001*
Dementia	1415(2.2%)	1206(3.7%)	<0.001*
Peripheral vascular disease	624(1.0%)	385(1.2%)	0.001*

Data are presented as n and percentage. * *p*-value< 0.05 was considered statistically significant after test.

Table S3. Risk of TKA in patients with and without fracture according to age, sex, and index year at a 1:2 ratio.

.	Age/sex/index year matching (1:2)	
	Fracture	
	Yes	No
Patient numbers	32,627	65254
TKA cases	432	855
Person-years	219,461	497,953
Incidence rate[a]	2.0	1.7
Univariate model		
crude HR (95% CI)	1.09 (0.97–1.23)	1 (ref.)
p value	0.143	
Multivariate model[b]		
aHR (95% CI)	1.19 (1.06–1.34)	1 (ref.)
p value	0.004*	

aHR, adjusted hazard ratio; CI, confidence interval; HR, hazard ratio; ref, reference. [a] Per 1000 person-years. [b] Multivariate Cox proportional hazard regression model with adjustment for all baseline characteristics shown in Table 1.

Table S4. Subgroup analysis of Cox's regression model for the association between fracture and TKA according to age, sex, and index year at a 1:2 ratio.

Variables	Age/sex/index year matching (1:2)				
	**Crude HR[a] (95% CI)**	***p*-value**	**Adjusted HR[a] (95% CI)**	***p*-value**	***p* for Interaction**
Main model					
Control	1.00		1.00		
Fracture	1.09 (0.97–1.23)	0.143	1.19 (1.06–1.34)	0.004*	
Age					
20-65 y/o					
Control	1.00		1.00		
Fracture	1.71 (1.41-2.08)	<0.001*	1.64 (1.35-2.00)	<0.001*	<0.001*
≧65 y/o					
Control	1.00		1.00		
Fracture	0.95 (0.82-1.10)	0.486	0.99 (0.85-1.15)	0.863	
Gender					
Male					
Control	1.00		1.00		
Fracture	1.33 (1.07-1.67)	0.012*	1.53 (1.22-1.92)	<0.001*	0.024*
Female					
Control	1.00		1.00		
Fracture	1.02 (0.88-1.17)	0.825	1.09 (0.95-1.25)	0.222	

CI, confidence interval; HR, hazard ratio. [a] Cox's proportional hazards model.
